# Ascites—IV

**Published:** 1909-07-03

**Authors:** 


### Medicine.

### ASCITES—IV.

DIFFERENTIAL DIAGNOSIS (*continued*).

4. *Distension Associated with Obesity.*—The abdomen may be uniformly and enormously distended as a part of general obesity, the mesentery, omentum, and abdominal wall being inches thick with fat. Here, satisfactory physical examination is very difficult. It may be impossible to determine with any degree of certainty the presence of a small or even a moderate amount of fluid in the peritoneal cavity. The abdomen is usually a little flattened in front, with a considerable amount of bulging in the flanks as the patient lies upon his back. The thickness of the abdominal wall may be felt. Deeper palpation is however very difficult, and tumours and enlarged organs may not be detected. A thrill is difficult to obtain even when much ascitic fluid is present.

5. *An Enormously Distended Bladder.*—This may reach to between the umbilicus and the ensiform cartilage causing general abdominal distension. But there is no bulging of the flanks. Careful palpation may reveal a rounded, tense, cyst-like tumour rising from the pelvis. It is in contact with the abdominal wall, and so yields a dull note on percussion in front, with a convex upper border to the dulness, and resonance in the flanks. In a woman ovarian cyst or pregnancy are more likely to suggest themselves as alternatives to distended bladder than is ascites. The condition is most commonly due to enlargement of the prostate in man, in which case the age helps the diagnosis greatly; in woman probably its commonest cause is retroversion of the gravid uterus.

If there is any question of such a condition being present a catheter should be passed and the urine drawn off, preferably by the medical attendant himself, since in cases of the kind the importance of avoiding every possible source of error is great.

6. *Phantom Tumour.*—In hysterical women, especially about the time of the menopause, the abdomen may become distended and simulate ascites, pregnancy, or ovarian cyst. The abdomen may be uniformly big, and sometimes enormous. The flanks are not bulged, the central part of the abdomen being most prominent, often with a transverse depression just below the ribs and above the pubes. The lumbar region of the spine may be found unduly arched forward.

On palpation what appears to be a smooth round regular tumour, which may even seem to be moveable, may be felt. There is neither thrill nor fluctuation, however. The abdominal wall may be tense from firm contraction of the recti. On percussion a resonant note is obtained all over the abdomen, but in some instances the degree of resonance appears to be impaired. A vaginal examination does not reveal any of the signs of ascites, pregnancy, or ovarian tumour. Under the influence of an anæsthetic the abdominal wall becomes flaccid, the swelling disappears, and nothing abnormal can be found on then making a careful examination of the abdomen. The importance of an examination under an anæsthetic cannot be overestimated, and in any doubtful cases it should be resorted to before laparotomy.

7. *Hydronephrosis.*—An enormous hydronephrosis has been known to cause great abdominal distension, and to be mistaken for ascites or ovarian cyst. In hydronephrosis close inspection may show the abdomen one side to be more prominent than the other, whereas in ascites the abdominal distension is usually general, both sides being equally enlarged. A local collection of fluid in the peritoneal cavity shut off by adhesions as a result of chronic peritonitis is more likely to be confused with hydronephrosis than free fluid in the peritoneal cavity is.

The hydronephrosis must be very large indeed if the tumour extends across the median line of the abdomen.

In hydronephrosis the opposite side may be resonant to percussion and the front of the abdomen dull, whereas in ascites both loins are usually dull and the front of the abdomen resonant. In the case of hydronephrosis the patient may have noticed that the swelling of the abdomen began in one loin and gradually increased in size. There may have been a history of renal colic and of hæmaturia. The size of the tumour may be found to vary from time to time, a diminution in size being associated with a considerably increased flow of urine. In some cases the colon may be felt or seen to lie over the front of the hydronephrosis, or percussion may discover the corresponding band of resonance running down the front of an otherwise dull tumour.

8. *Pancreatic Cyst.*—A large pancreatic cyst, or more properly speaking a cyst of the lesser omental sac with the pancreas incorporated in its wall, may cause general abdominal distension. A careful inspection of the abdomen will show that the most prominent part is between the umbilicus and the ensiform cartilage, and that the greatest circumference is above the level of the umbilicus.

On account of the deep origin of the cyst and its close proximity to the aorta, a well-marked transmitted pulsation may be seen and felt in the upper part of the abdomen. The stomach is usually lying in front of a small pancreatic cyst, but when the latter attains an enormous size the stomach becomes pushed upwards and the transverse colon downwards. On percussion the flanks are resonant; if the stomach is distended with gas and it is lying in front of the cyst, a resonant note may be obtained on percussion over it. More often, owing to the displacement of stomach and colon by a cyst big enough to cause general abdominal distension, the front of the cyst is dull to percussion, with tympanitic resonance above and below it.

Increase in size of the abdomen may be noticed by the patient to begin above the umbilicus. In some cases the stools are fatty, and the urine contains sugar.

(9) *Enormous Enlargement of the Liver, or Enormous Enlargement of the Spleen*, may cause general abdominal distension. A careful physical examination should, however, enable one in either case to determine that the enlargement is due to the presence of a solid mass which comes down from under the costal margin above, though it may extend below even so far as the pubes; that there is neither fluctuation nor a thrill; that on percussion there is dulness which extends upwards and is continuous with either the normal hepatic or splenic dulness; and that a well-defined edge may be felt below, extending in the case of the liver from the right below obliquely upwards towards the left, whilst a well-marked notch or notches in it may be almost pathognomonic of the spleen. The only variety of splenic enlargement likely to cause real difficulty when physical examination alone is relied upon is that of splenomedullary leucæmia; here the blood-count will clear up any doubts. The only remaining difficulty may be when ascites coincides with enlargement of the viscus. In these circumstances the sign of "dipping" often becomes one of the greatest value.

(10) *Enormous Dilatation of the Stomach* may cause marked abdominal distension which at first sight may appear to be general. A careful physical examination, however, may reveal that the distension is chiefly to the left of or below the umbilicus; that the epigastric region is flattened; that the outline of the lesser and greater curvatures of the stomach may be visible in some cases; that a well-marked peristaltic wave may be seen to travel from left to right from time to time, and in some cases this may further be watched returning from right to left; that a tympanitic note is elicited on percussion; that a *bruit d'airain* is obtainable over the viscus with the aid of coin-tap and stethoscope; that a well-marked succussion splash may be obtained, indicating the presence of fluid and gas in a closed sac—a physical sign which is never obtained in ascites.

